# Variation in the repulsive guidance molecule family in human populations

**DOI:** 10.14814/phy2.13959

**Published:** 2019-02-11

**Authors:** Peter Rotwein

**Affiliations:** ^1^ Department of Biomedical Sciences Paul L. Foster School of Medicine Texas Tech Health University Health Sciences Center El Paso Texas

**Keywords:** Genomics, hemochromatosis, hemojuvelin, human variation, population genetics, repulsive guidance molecule, RGMA, RGMB, RGMC

## Abstract

Repulsive guidance molecules, RGMA, RGMB, and RGMC, are related proteins discovered independently through different experimental paradigms. They are encoded by single copy genes in mammalian and other vertebrate genomes, and are ~50% identical in amino acid sequence. The importance of RGM actions in human physiology has not been realized, as most research has focused on non‐human models, although mutations in RGMC are the cause of the severe iron storage disorder, juvenile hemochromatosis. Here I show that repositories of human genomic and population genetic data can be used as starting points for discovery and for developing new testable hypotheses about each of these paralogs in human biology and disease susceptibility. Information was extracted, aggregated, and analyzed from the Ensembl and UCSC Genome Browsers, the Exome Aggregation Consortium, the Genotype‐Tissue Expression project portal, the cBio portal for Cancer Genomics, and the National Cancer Institute Genomic Data Commons data site. Results identify extensive variation in gene expression patterns, substantial alternative RNA splicing, and possible missense alterations and other modifications in the coding regions of each of the three genes, with many putative mutations being detected in individuals with different types of cancers. Moreover, selected amino acid substitutions are highly prevalent in the world population, with minor allele frequencies of up to 37% for RGMA and up to 8% for RGMB. These results indicate that protein sequence variation is common in the human RGM family, and raises the possibility that individual variants will have a significant population impact on human physiology and/or disease predisposition.

## Introduction

The repulsive guidance molecule (RGM) family consists of three members, *RGMA*,* RGMB*, and *RGMC* (also known as *HFE2* and *HJV*) (Monnier et al. 2002; Kuninger et al. 2004; Niederkofler et al. 2004; Papanikolaou et al. 2004; Samad et al. 2004; Schmidtmer and Engelkamp 2004), that are encoded by single‐copy genes in human and other vertebrate genomes (Severyn et al. 2009). The family received its name from a then‐novel axonal guidance molecule termed RGM that was characterized in 2002 (Monnier et al. 2002). Subsequent studies identified two related proteins in mammals, termed RGMB and RGMC (Papanikolaou et al. 2004; Samad et al. 2004; Schmidtmer and Engelkamp 2004), and fourth member in teleosts, called RGMD (Corradini et al. 2009; Siebold et al. 2017). The original RGM is now named RGMA (Corradini et al. 2009; Severyn et al. 2009; Siebold et al. 2017).

RGMA and RGMB have been shown to be expressed in the central nervous system during development (Schmidtmer and Engelkamp 2004), and their discoveries indicated that they were involved in controlling axonal patterning and neuronal survival (Monnier et al. 2002; Matsunaga et al. 2004; Niederkofler et al. 2004; Rajagopalan et al. 2004; Samad et al. 2004). In contrast, RGMC was initially characterized through its gene, which was found within a locus that was linked to a severe form of an iron storage disease that primarily affects children, termed juvenile hemochromatosis (Papanikolaou et al. 2004). The gene was termed *HFE2* after *HFE* (high iron [chemical symbol Fe]), the initial gene whose mutations were found in hemochromatosis (Papanikolaou et al. 2004). The encoded protein, RGMC, is also called hemojuvelin (HJV), because of its relationship with juvenile hemochromatosis (Papanikolaou et al. 2004). Unlike RGMA and RGMB, RGMC/HFE2/HJV is produced in the liver and in cardiac and skeletal muscle, and not within the nervous system (Kuninger et al. 2004; Papanikolaou et al. 2004; Schmidtmer and Engelkamp 2004).

RGMA, RGMB, and RGMC are glycosylphosphatidylinositol (GPI) ‐linked cell membrane‐associated glycoproteins (Corradini et al. 2009; Severyn et al. 2009; Siebold et al. 2017), and the paralogs share ~50% amino acid identity and several structural motifs, including 14 cysteine residues in comparable locations within the three proteins (Corradini et al. 2009; Severyn et al. 2009; Siebold et al. 2017). All three RGMs also appear to undergo a series of similar biosynthetic and processing steps leading to both cell‐associated and soluble protein species (Babitt et al. 2005; Samad et al. 2005; Kuninger et al. 2006). All three proteins also interact with members of the bone morphogenetic protein (BMP) family, where they function as co‐receptors (Core et al. 2014). BMPs are members of the transforming growth factor‐*β* (TGF‐*β*) super‐family, and play key roles in different developmental and cell fate decisions (Hata and Chen 2016; Morikawa et al. 2016; Siebold et al. 2017). BMPs bind as dimers to specific type I and type II serine/threonine kinase receptors, and initiate a protein kinase cascade which culminates in the activation by serine phosphorylation of Smads 1, 5, and 8, signal transducers and transcription factors that regulate the expression of many BMP‐dependent target genes (Hata and Chen 2016; Morikawa et al. 2016).

RGM proteins also bind to the cell surface trans‐membrane molecule, neogenin (Matsunaga et al. 2004, 2006; Rajagopalan et al. 2004; Kuns‐Hashimoto et al. 2008; Yang et al. 2008), a member of the netrin‐binding, deleted in colon cancer family, which also includes DCC and UNC5 (Keino‐Masu et al. 1996; Leonardo et al. 1997; Mehlen and Mazelin 2003; Bernet and Mehlen 2007). The actions of RGMA in both neuronal guidance and neuronal survival are mediated by neogenin (Matsunaga et al. 2004; Conrad et al. 2007). The other RGM proteins also can bind to neogenin, but it does not appear to play the predominant role in their biological actions (Kuns‐Hashimoto et al. 2008; Xia et al. 2008; Yang et al. 2008; Corradini et al. 2009; Siebold et al. 2017).

Major recent advances in human genetics and genomics now present distinct opportunities for improving our knowledge of human physiology and disease susceptibility, and for gaining new insights into human variation, human origins, and evolution (Acuna‐Hidalgo et al. 2016; Katsanis 2016; Quintana‐Murci 2016; Battle et al. 2017; eGTEx Project, 2017). Here I use the RGM family to show how to understand and integrate this information, by accessing publically available genomic and gene expression repositories to examine human *RGM* genes in detail. Results reveal extensive variation in gene expression patterns, substantial alternative RNA splicing, and a range of possible missense alterations and other modifications in the coding regions of each of the three genes. Taken together, these observations will provide new opportunities to define the dynamics and range of RGM actions in different physiological and pathological contexts, and will serve as a template and guide that can be applied to other gene families in humans and other species.

## Methods

### Databases and analyses

Information on human *RGMA*,* RGMB*, and *RGMC* (*HFE2*/*HJV*) loci and genes was obtained from the Ensembl (www.ensemble.org) and UCSC Genome Browsers (https://genome.ucsc.edu), by searching genome assembly, GRCh38, with each gene name. The different classes of transcripts for each gene were also derived from the Ensembl and UCSC browsers. Data on levels of *RGMA*,* RGMB*, and *RGMC* mRNAs in human tissues were extracted from the Genotype‐Tissue Expression project (GTEx) portal (Battle et al. 2017) (https://www.gtexportal.org/) by searching the “transcriptome” menu with the name of each gene. Relative levels of specific mRNA isoforms were calculated from primary information within the “exon expression” sub‐menu of GTEx. Human RGMA, RGMB, and RGMC protein sequences were isolated from the National Center for Biotechnology Information (NCBI) Consensus CDS Protein Set (https://www.ncbi.nlm.nih.gov/CCDS/). Information on predicted population variation in these three proteins was obtained from the Exome Aggregation Consortium (ExAc) genome browser (http://exac.broadinstitute.org/), by examining the primary data from each gene after it was downloaded as a series of CSV files. ExAc contains results of sequencing the exons of 60,706 individuals (Karczewski et al. 2017). Data on predicted alterations in RGMA, RGMB, and RGMC proteins in different cancers were extracted from the cBio portal for Cancer Genomics (http://www.cbioportal.org/), which lists gene alterations from 65,690 different individuals from 225 cancer studies (Cerami et al. 2012; Gao et al. 2013), and from the National Cancer Institute Genomic Data Commons data portal (https://portal.gdc.cancer.gov/), which contains analogous information on 32,555 cancer cases.

## Results

### Topography of human RGM loci

**Figure 1:**
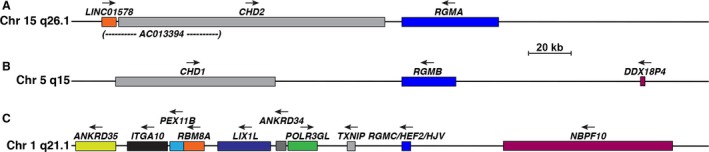
Organization of human *RGM* loci. (A) Map showing the human *RGMA* locus on chromosome 15q26.1. Genes include *long intergenic non‐protein coding RNA 1578* (*LINC01578*), *clone‐based (Ensembl) gene AC012294.1*,* chromo‐domain helicase DNA binding protein 2* (*CHD2*), and *RGMA*. (B) Illustration of the human *RGMB* locus on chromosome 5q15. Genes include *chromo‐domain helicase DNA binding protein 1* (*CHD1*), *RGMA*, and *DEAD‐box helicase 18 pseudogene 4* (*DDX18P4*). (C) Map showing the human *RGMC*/*HFE2/HJV* locus on chromosome 1q21.1. Genes include the following: ankryin repeat domain 35 (*ANKRD35*), integrin subunit alpha 10 (*ITGA10*), peroxisomal biogenesis factor 11 beta (*PEX11B*), RNA binding motif protein 8A (*RBM8A*), limb and CNS expressed 1 like (*LIX1L*), ankryin repeat domain 34A (*ANKRD34A*), RNA polymerase III subunit G like (*POLR3GL*), thioredoxin interacting protein (*TXNIP*), *RGMC*/*HFE2/HJV*, NBPF member 10 (*NBPF10*). For A–C, the scale bar represents 20 kb, and a horizontal arrow indicates the direction of transcription for each gene.

The three single‐copy human *RGM* genes reside on different autosomes. Three other genes are found within the 300 kb segment of chromosome 15q26.1 containing *RGMA* (Fig. 1A), and the locus is conserved with both mouse and chicken genomes (Severyn et al. 2009). *RGMB* on human chromosome 5q15 is also a part of a chromosomal region with conserved synteny with mouse and chicken genomes (Severyn et al. 2009), but only two genes, *CHD1* and *DDX18P4*, are found within the 300 kb region depicted in Figure 1B. Of note, the paralogous relationship between adjacent genes on both chromosomes, *CHD2* and *RGMA* on chromosome 15 and *CHD1* and *RGMB* on chromosome 5, and their shared convergent transcriptional orientation indicates that these loci were generated by segmental duplication (Severyn et al. 2009). By contrast with the chromosomal regions of *RGMA* or *RGMB*, the *RGMC/HFE2/HJV* locus on human chromosome 1q21.1 is far more gene dense (Fig. 1C), and contains nine other genes that also are present in the orthologous mouse locus (Severyn et al. 2009).

### RGM gene structures and expression patterns in human tissues

**Figure 2:**
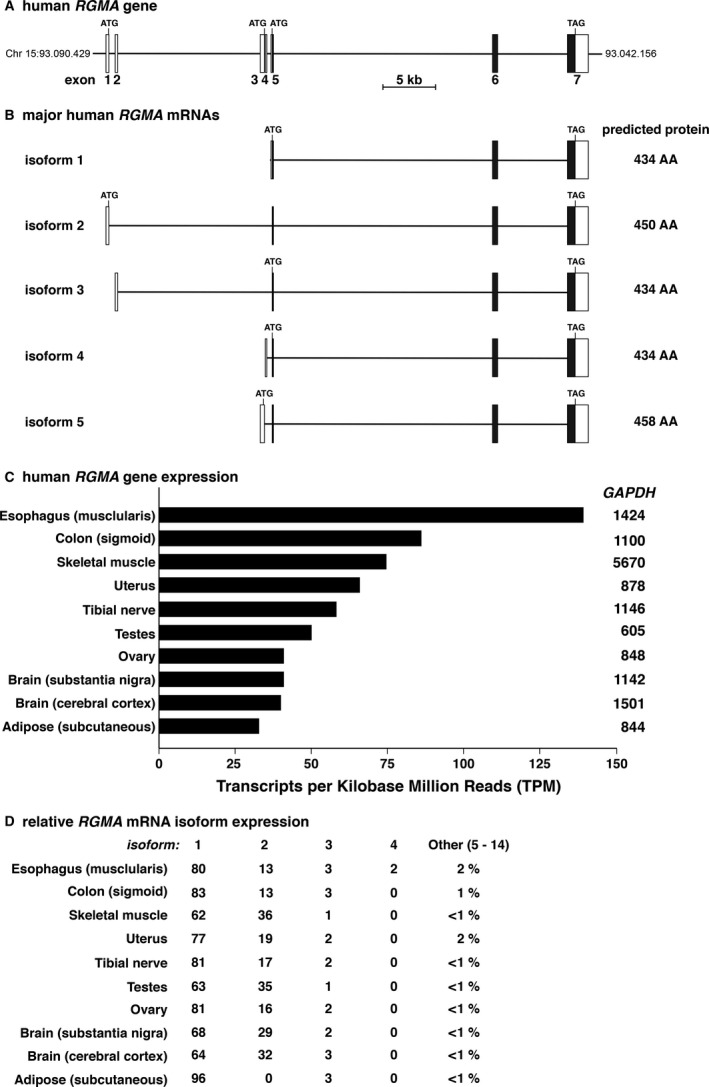
Human *RGMA* gene structure and expression. (A) Schematic of the human *RGMA* gene, illustrating exons 1–7, and ATG and TAG codons. Exons are represented as boxes, with coding regions in black and non‐coding segments in white, and introns as horizontal lines. A scale bar is shown. (B) Diagrams of the four major classes of human *RGMA* mRNAs represent the following transcripts from the Ensembl genome browser: isoform 1, ENST00000543599.5; isoform 2, ENST00000329082.11; isoform 3, ENST00000542321.6; and isoform 4, ENST00000425933.6. The protein encoded by each transcript is listed to the right of each diagram. (C) RGMA gene expression in 10 different human tissues and organs. Data were obtained from the GTEx portal, and are graphed as the mean number of transcripts per kilobase million reads (TPM), with the mean transcript abundance of glyceraldehyde 3‐phosphate dehydrogenase (GAPDH) listed to the right of each *RGMA* RNA level. The number of samples for each organ and tissue is as follows: esophagus (370), sigmoid colon (233), skeletal muscle (564), uterus (111), tibial nerve (414), testes (259), ovary (133), substantia nigra (88), cerebral cortex (158) and subcutaneous adipose tissue (42). (D) Relative expression of *RGMA* mRNAs in 10 different human organs and tissues (%) from the GTEx database. Transcripts 1–5 are illustrated in part B above, and mRNAs 6–14 are found in GTEx.

The human *RGMA* gene spans ~45 kb of chromosomal DNA and consists of seven exons that are used in the vast majority of transcripts reported within the human Genotype‐Tissue Expression project (GTEx) (Battle et al. 2017; eGTEx Project, 2017) (Fig. 2). The five predominant *RGMA* mRNA isoforms described in GTEx consist of either 3 or 4 exons, and encode one of three very similar RGMA proteins of 434, 450, or 458 amino acids, with all differences being located at the NH_2_‐termini of the proteins (Fig. 2B). *RGMA* mRNAs are expressed in 48 of the 51 different human organs and tissues found in the GTEx portal. The 10 organs and tissues with the highest abundance of *RGMA* mRNAs include esophagus, colon, skeletal muscle, uterus, tibial nerve, testes, ovary, several brain regions, and adipose tissue (range of expression from 139 to 32 transcripts per kilobase million reads in order (TPM; Fig. 2C). By contrast, glyceraldehyde 3‐phosphate dehydrogenase (GAPDH), a typical “control” transcript in gene expression studies was 10–70‐times more abundant than RGMA in the organs and tissues examined here (Fig. 2C). The vast majority of *RGMA* mRNAs found in human tissues according GTEx comprised of isoforms 1 or 2 (~93–99% of all transcripts; Fig. 2D). In contrast, the major RGMA protein in the Exome Aggregation Consortium (ExAC) gene dataset is predicted to have 458 amino acids, which is encoded by isoform 5 in Figure 2B. This mRNA is expressed minimally in the ten human tissues catalogued by GTEx and presented here (Fig. 2D, and see below).

**Figure 3:**
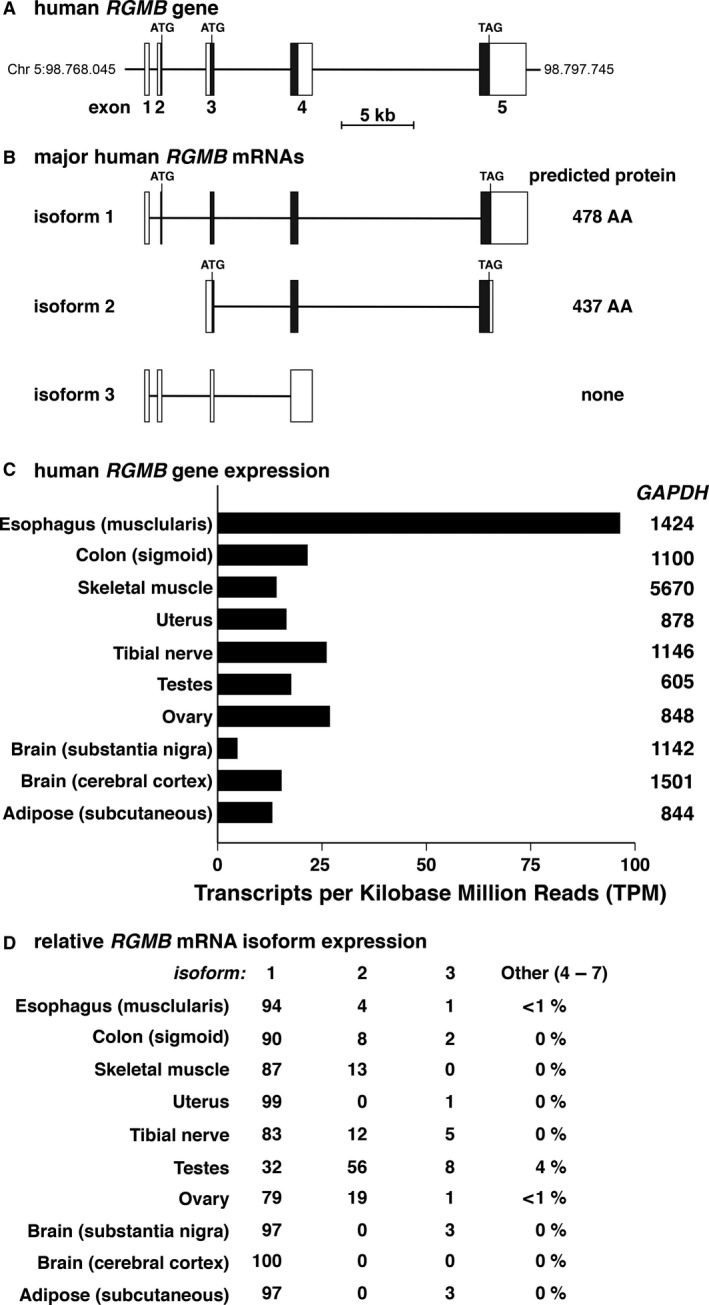
Human *RGMB* gene structure and expression. (A) Schematic of the human *RGMB* gene, depicting exons 1–5, and ATG and TAG codons. Exons are represented as boxes, with coding regions in black and non‐coding segments in white, and introns as horizontal lines. A scale bar is shown. (B) Diagrams of the three major classes of human *RGMB* mRNAs represent the following transcripts from the Ensembl genome browser: isoform 1, ENST00000308234.11; isoform 2, ENST00000513185.1; and isoform 3, ENST00000434027.2. The protein encoded by each transcript is listed to the right of each diagram. (C) *RGMB* gene expression in 10 different human tissues and organs. Data were obtained from the GTEx portal, and are graphed as TPM. Mean transcript abundance of GAPDH is listed to the right of each *RGMB* RNA level. (D) Relative expression of *RGMB* mRNAs in 10 different human organs and tissues (%) in the GTEx database. Transcripts 1–3 are illustrated in part B above, and mRNAs 4–7 are found in GTEx.

The human *RGMB* gene is slightly more compact than *RGMA*, and its five major exons extend over ~26 kb of genomic DNA (Fig. 3A). The three predominant transcripts in GTEx also are derived by alternative RNA splicing, but only two of these mRNAs appear to encode RGMB proteins of either 478 or 437 amino acids (Fig. 3B). *RGMB* transcripts are expressed in 49 of the 51 different organs and tissues found in GTEx; except for esophagus, mRNA levels are 2–3‐fold lower than for *RGMA* mRNAs (compare Figs. 3C, 2C). The majority of expressed *RGMB* mRNAs encode RGMB proteins, primarily the 478 amino acid species (isoform 1, Fig. 3D).

**Figure 4:**
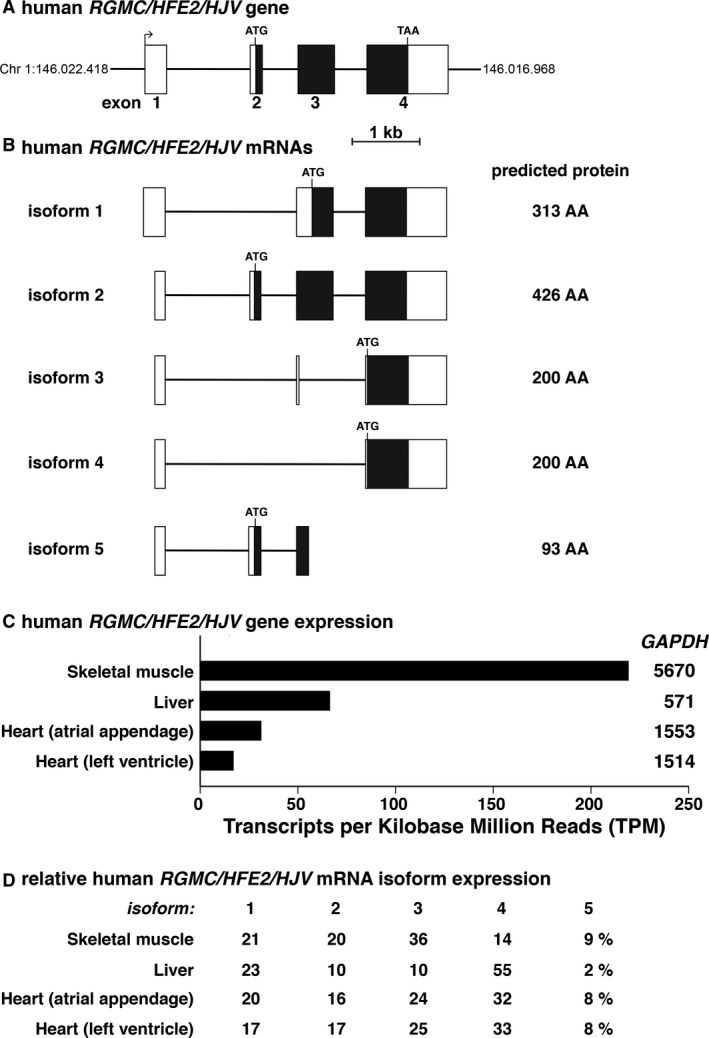
Human *RGMC*/*HFE2/HJV* gene structure and expression. (A) Schematic of the human *RGMC*/*HFE2/HJV* gene, illustrating exons 1‐4, and ATG and TAA codons. Exons are depicted as boxes, with coding regions in black and non‐coding segments in white, and introns as horizontal lines. A scale bar is shown. (B) Diagrams of the five major classes of human *RGMC*/*HFE2/HJV* transcripts represent the following mRNAs from the Ensembl genome browser, respectively: isoform 1, ENST00000497365.5; isoform 2, ENST00000357836.5; isoform 3, ENST00000336751.10; isoform 4, ENST00000475797.1; and isoform 5, ENST00000421822.2. The protein encoded by each transcript is listed to the right of each diagram. (C) *RGMC*/*HFE2/HJV* gene expression in different human tissues and organs. Data were obtained from the GTEx portal, and are graphed as TPM. Mean transcript abundance of GAPDH is listed to the right of each *RGMC*/*HFE2/HJV* RNA level. The number of samples for each organ and tissue is as follows: skeletal muscle (564), liver (175), atrial appendage (297), and left ventricle (303). (D) Relative expression of *RGMC*/*HFE2/HJV* mRNAs in different human organs and tissues (%) found in the GTEx database.

Human *RGMC/HFE2/HJV* at ~4.5 kb in length is substantially smaller than either *RGMA* or *RGMB*, and is composed of four exons and three introns (Fig. 4A). There are five major transcripts expressed in human organs and tissues, and they encode proteins of variable lengths, from 93 to 426 amino acids (Fig. 4B). Unlike *RGMA* or *RGMB*,* RGMC/HFE2/HJV* mRNAs can be detected only in human skeletal muscle, liver, and heart, and were found at steady‐state levels that were 9–25‐fold less abundant than GAPDH (Fig. 4C). Perhaps surprisingly, the *RGMC/HFE2/HJV* mRNA that encodes the full‐length 426‐residue RGMC/HJV protein (isoform 2) comprises only 10–20% of transcripts in human organs and tissues according to GTEx (Fig. 4D). The reasons for the low level of gene expression for isoform 2 are unknown, but could reflect differential RNA stability, or the technical conditions under which the tissues were obtained and RNA samples isolated and processed (see Discussion).

### Predicted variation in RGM proteins in human populations

ExAC contains DNA sequence information from the exons of genes from 60,706 people representing different population groups from around the world (Bahcall 2016; Lek et al. 2016; Ruderfer et al. 2016; Karczewski et al. 2017). The data have revealed substantial variation within the coding regions of genes in this large population, but also showed that most alterations were uncommon, as the majority was detected in a single allele, and over 99% were found in <1% of the study group (Lek et al. 2016). Most of this previously described variation consists of synonymous nucleotide changes and amino acid substitutions (Lek et al. 2016).

**Figure 5:**
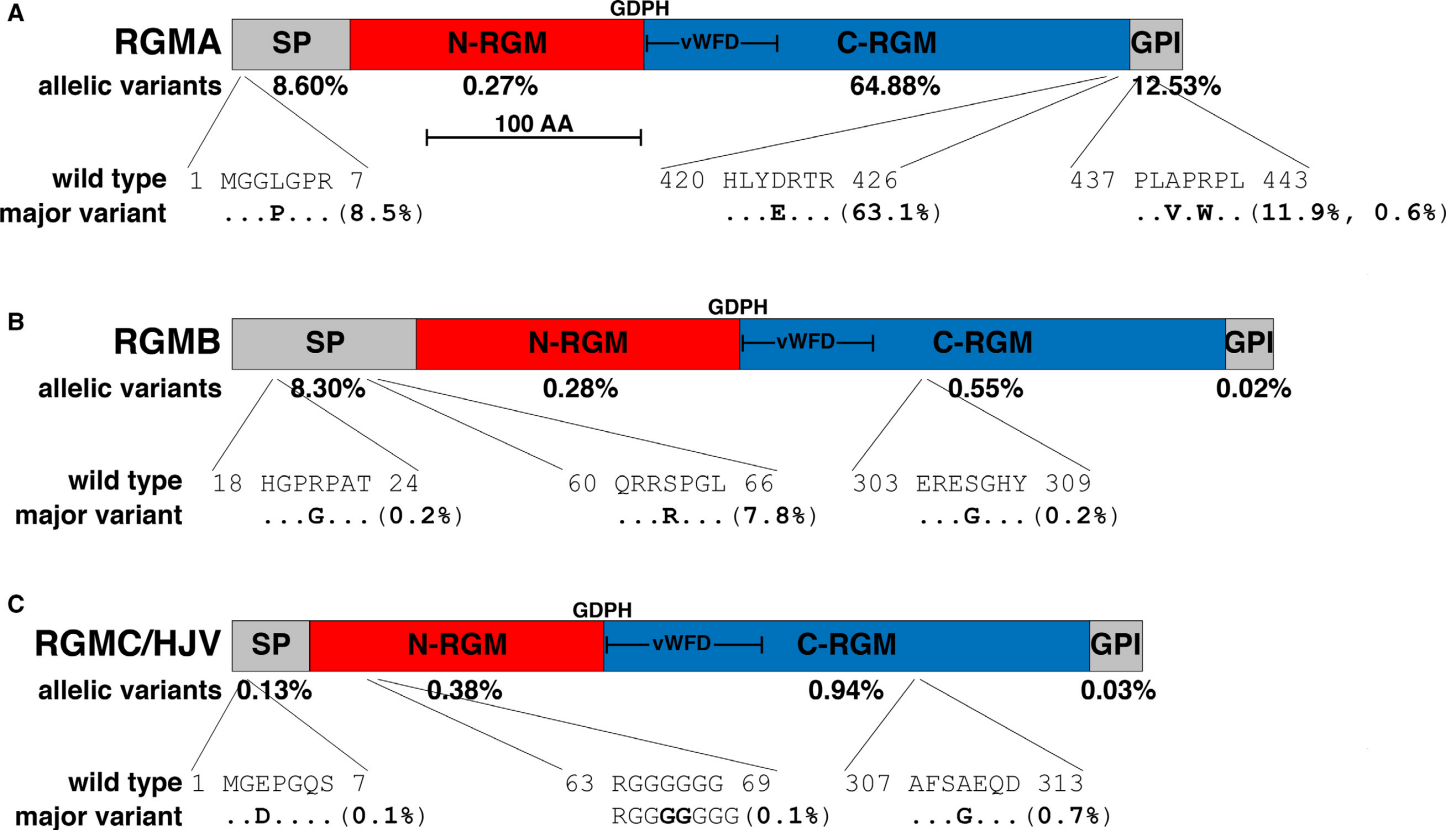
Population variation in human RGM proteins. (A–C) The three human RGM protein precursors are composed of four identifiable regions, termed the signal peptide (SP), N‐terminal RGM (N‐RGM domain), C‐terminal RGM (C‐RGM segment), which includes a partial von Willebrand factor type D domain (vWFD), and the glycosylphosphatidylinositol recognition sequence (GPI), which is cleaved as part of the biosynthetic steps leading to glycosylphosphatidylinositol addition to the maturing protein (Lebreton et al. 2018). The scale bar represents 100 amino acids. The location of the GDPH autocatalytic cleavage site (Siebold et al. 2017) is listed above each diagram. (A) Human RGMA highlighted by ExAC consists of a 458‐residue protein. The overall population prevalence of variant alleles for each segment of the protein is listed below the map, and the most common variants are illustrated in single letter amino acid code. (B) The human RGMB found in ExAC consists of 478‐amino acids. The population prevalence of variant alleles for each segment of the protein is listed below the map, and the most common variants are depicted in single letter amino acid code. (C) Human RGMC/HJV consists of a 426‐residue protein in ExAC. The overall population prevalence of variant alleles for each segment of the protein is listed below the map, and the most common variants are shown in single letter amino acid code.

Examination of RGM family members in ExAC revealed a wide range of potential alterations in their exons, with most of the predicted changes consisting of missense mutations (92–96% of modified alleles, depending on the gene, Table 1). Second most common were changes in the reading frame, including inserted stop codons (1–7%, Table 1). Overall, the total number of different allelic variants per gene was similar for all RGM family members, and ranged from 143 for *RGMB* to 185 for *RGMA*, but their population frequency varied by a factor of 60, from 1.4% for *RGMC/HFE2/HJV* to 86% for *RGMA*, with the vast majority of changes being accounted for just a few modifications (Fig. 5). As 99.1% of missense alleles were detected in ≤1% of the ExAC study population, overall results regarding the frequency of differences in the human RGM family proteins are consistent with the general conclusions from ExAC (Lek et al. 2016), with the exception of the few highly prevalent allelic variants depicted in Figure 5.

**Table 1:** Human population variation in RGMA, RGMB, and RGMC

Protein	Number of codons^1^	Missense and in‐frame insertions‐deletions	Frame shifts; stop codons	Splicing site changes	Loss of start codon	Loss of stop codon	Total number of different changes	Variants occurring once	Total variant alleles in population
RGMA	458	178	2	5	0	0	185	96	86.0%
RGMB	478	134	6	3	0	0	143	78	9.1%
RGMC	426	157	12	1	1	0	171	109	1.4%

1 Based on transcripts used in ExAC database. All RGMB and RGMC variants mapped to the 478 or 426 codons, respectively, corresponding to a full‐length protein. For RGMA, 17 variants were not counted as they mapped to a transcript corresponding to a smaller predicted protein of 61 residues that undergoes rapid decay.

### Population variation in RGMA

Alterations in *RGMA* have not been linked to date with the pathogenesis of any specific human diseases. Thus, the functional consequences of three prevalent specific amino acid substitutions in the RGMA protein (Leu^4^ to Pro in the signal peptide (8.5% in the population), the conservative substitution of Asp^423^ to Glu in the C‐terminal RGM domain (63.1%), and Ala^439^ to Val in the GPI‐anchor segment (11.9%), Fig. 5A) in either human physiology or pathology are not known.

As with some other proteins, a large number of alterations in *RGMA* have been found to be associated with a variety of different cancers, according to the analysis of data in the cBio portal for Cancer Genomics (Table 2) and the National Cancer Institute Genomic Data Commons portal, although the functional consequences are unknown. Potential mutations at 78 different locations in *RGMA* coding exons have been detected in 38 different neoplasms, with the prevalence of these changes ranging from 3.5% in ovarian cancer and 2.8% in esophageal, gastric, and small cell lung cancer, and in soft tissue sarcoma, to <0.3% in prostate and renal carcinoma, various leukemias and lymphomas, and others (see cancer type in: http://www.cbioportal.org/index.do?session_id=5b5f49be498eb8b3d5672991). The vast majority of alterations consisted of amino acid substitutions (76 different modifications at 71 different sites; Table 2), of which 43 at 32 locations were present in the ExAC population, although generally at low frequency (Table 2). However, three of the cancer‐associated amino acid substitutions were among the more common allelic variants in the population (Leu^4^ to Pro, 8.5% of ExAC alleles, Ala^439^ to Val, 11.9%, and Arg^441^ to Trp, 0.6%; Fig. 5A), and thus may have been detected by chance rather than through disease association. However, the most prevalent allele in ExAC, Asp^423^ to Glu, seen in 63.1% of the population (Fig. 5A), was absent in any of the cancer studies compiled here (Table 2). Other changes associated with different neoplasms included premature stop codons and frame‐shifts, none of which were found in ExAC (seven examples, Table 2).

**Table 2:** Cancer‐associated mutations in RGMA^1^

Mutation	Population variant	ExAC prevalence
L4P	L4P	10396 alleles
L16V	None	–
R21P	None	–
M27I	None	–
G30E	None	–
S34stop	None	–
F38L	F38S	1 allele
P40S	None	–
A43D	A43V	1 allele
F44L	F44C	3 alleles
P55L	P55L	15 alleles
G71C	None	–
D79G	D79N, D79Y	1, 1 allele
P81L	None	–
R88H	R88H	1 allele
R95L, R95Q, R95W	R95Q	7 alleles
R96W	R96Q, R96W	3, 6 alleles
T97M	None	–
D104N	None	–
H108N	None	–
S119T	None	–
R133C	R133G, R133H	5, 8 alleles
R135H	R135C, R135H	4, 5 alleles
P139L	P239S, P139T	21, 143 alleles
E145Q	None	–
E151K	E151K	1 allele
E156K	None	–
P164S	None	–
H170Tfs34stop	None	–
G175R	G175R	1 allele
T181I	None	–
T189I	None	–
P196L	None	–
N204S	N204K	1 allele
P211H	None	–
A217V	A217V	3 alleles
Q231R	None	–
E233A, E233K	None	–
P248S	P248L	1 allele
V252L	V252M	5 alleles
K256stop	None	–
S266N	None	–
E271K, E271stop	None	–
A281D	None	–
V290M	None	–
R292C	None	–
V302A, V302I	V302I	13 alleles
M304I	None	–
V309A	None	–
E313K	None	–
W315L	W315G	1 allele
R325W	R325Q, R325W	1, 4 alleles
G326V	None	–
G346S	G346S	1 allele
R348H	R348C, R348H	15, 42 alleles
L350M	None	–
A353T	A353T	2 alleles
P357S	None	–
E3613Rfs36^1^	None	–
V369M	V369L, V369M	6, 1 alleles
C372stop	C372R, C372Y	1, 1 alleles
V378A	V378M	1 allele
E379stop	None	–
V387I	None	–
D389N	None	–
D395N	None	–
V396M	None	–
A402T	None	–
V404M	None	–
A405V	None	–
L406F	None	–
L412I	None	–
P429L	None	–
A432V	A432V	4 alleles
A439V	A439G, A439G	1, 14451 alleles
R441T	R441Q, R441W	34, 668 alleles
P442A	P442L	1 allele
A446T	A446P, A446T	1, 7 alleles

1 Amino acid positions modified to agree with ExAC assignments (see Text).

### Population variation in RGMB

Changes in *RGMB* also have not been connected to the pathogenesis of any human diseases to date. As with *RGMA*, the functional consequences to human physiology or pathology of the single predicted single amino acid substitution in *RGMB* that is prevalent in the ExAC population (Ser^63^ to Arg in the signal peptide (7.8%), Fig. 5B) are unknown.

Changes in *RGMB* also have been detected in a number of different cancers (Table 3), but as with *RGMA*, the possible functional impacts are not known. Potential mutations have been identified at 69 different locations in coding portions of the *RGMB* gene in 38 cancer studies, with the prevalence of these changes ranging from nearly 10% in prostate cancer and 7.5% in adrenocortical carcinoma to 0.3% or less in cervical, thyroid, bone, skin, and brain cancers, in leukemias and lymphomas, and in other neoplasms (see cancer type in: http://www.cbioportal.org/index.do?session_id=5b609381498eb8b3d5672df4). Most of the alterations consisted of amino acid substitutions (73 different modifications; Table 3), of which 26 at 19 sites were identified in ExAC at a frequency of 0.1–0.001% (Table 3), except for the highly prevalent Ser^63^ to Arg allele at 7.8% (Fig. 5B). The other 18 changes, which included both premature stop codons and frame‐shifts, which led to stop codons, were not found in ExAC (Table 3).

**Table 3:** Cancer‐associated mutations in RGMB^1^

Mutation	Population variant	ExAC prevalence
R49Kfs51stop	None	–
S63R	S63R	9454 alleles
Q90stop	None	–
A93Nfs14stop	A93T	1 allele
Q94H	None	–
R96Q	R96Q, R96stop	132, 1 alleles
S104R	None	–
V105E	None	–
H110P	None	–
E120stop	None	–
E121Vfs34stop	E121A, E121stop	1, 1 alleles
R127C	R127H	2 alleles
R135Q	R135Q	1 allele
C140Y	None	–
R141H	R141C, R141H	2, 11 alleles
N143S	None	–
V145L, V145_Y105insL	None	–
H147D	None	–
L151F	None	–
L156H	None	–
Q159H	None	–
R160M	None	–
G166stop	None	–
H183Qfs35stop	None	–
E190Tfs28stop	None	–
L206F	None	–
L230I	None	–
N233D	N233T	1 allele
N234del	None	–
V243I	V243E, V243I	8, 4 alleles
P244H	None	–
G248E	None	–
X257_splice		
A263G	None	–
C267Y	None	–
T268K	None	–
Y273stop	None	–
A283T	A283T	1 allele
G287D	None	–
G292R, G292V	None	–
R300H	R300C, R300H	4, 4 alleles
V302L, V302M	V302M	8 alleles
G307A	None	–
A314P	A314T, A314V	1, 2 alleles
R328C, R328H	R328C	1 allele
R335C	None	–
A341V	None	–
Q351K	None	–
E361K	None	–
L392Gfs9stop	None	–
Q398E	None	–
E401Q	None	–
P404S	None	–
Y409D	Y409C	1 allele
F415V	None	–
T420N	None	–
F425L	None	–
A428T	A428P, A428T	2, 5 alleles
L433Gfs9stop	None	–
E3434stop,	None	–
A438V	None	–
K443N	None	–
S451N	None	–
N454Kfs9stop	N454S	3 alleles
T456I	None	–
R458H	R458H	4 alleles
L463stop	L463F	2 alleles
T470Nfs33stop	None	–
L478stop	None	–

1 Amino acid positions modified to agree with ExAC assignments (see Text).

### Disease links and population variation in RGMC/HFE2/HJV

Unlike other members of the human RGM family, *RGMC/HFE2/HJV* was first characterized as the gene associated with the severe iron storage disease, juvenile hemochromatosis (Papanikolaou et al. 2004), and identification of mutations in the gene in affected individuals defined causality (Lanzara et al. 2004; Papanikolaou et al. 2004; Gehrke et al. 2005), which was confirmed by mouse gene knockout models (Huang et al. 2005; Niederkofler et al. 2005). The majority of over 40 different mutations that have been found in the individuals with juvenile hemochromatosis are amino acid substitutions, but more than a third predict truncated proteins because of introduced premature stop codons (Table 4). Almost half of these disease‐associated alleles can be found in the ExAC population, but nearly all are present at very low prevalences of 0.025–0.001% (Table 4). The only exception, Ala^310^ to Gly, is the most common *RGMC/HFE2/HJV* variant in ExAC, and has a population frequency of 0.7% (Fig. 5C).

**Table 4:** Juvenile hemochromatosis‐linked mutations in RGMC/HJV

Mutation	Population variant	ExAC prevalence
Q6H	Q6H	3 alleles
L27fs51stop	None	–
R54stop	None	–
G66stop	None	–
V74fs113stop	None	–
C80R	C80R	1 allele
S85P	None	–
G99R, G99V	None	–
L101P	L101P	1 allele
C119F	None	–
R131fs245stop	R131W	1 allele
D149fs245stop	None	–
L165stop	L165stop	1 allele
A168D	A168V	1 allele
F170S	None	–
D172E	D172E	1 allele
R176C	None	–
W191C	None	–
L194P	L194P	1 allele
N196K	None	–
S205R	None	–
I222N	I222M, I222N	1, 1 alleles
K234stop	None	–
D249H	None	–
G250V	None	–
N269fs311stop	N269S	1 allele
I281T	None	–
R288W, R288Y	R288Q, R288W	1, 2 alleles
E302K	E302D, E302K	1, 32 alleles
A310G	A310G	846 alleles
Q312stop	None	–
G319fs341stop	G319A	3 alleles
G320V	G320V, G320W	21, 2 alleles
C321W, C321stop	C321W, C321Y, C321stop	2, 1, 1 alleles
R326stop	R326Q, R326stop	5, 2 alleles
S328fs337stop	S328T	1 allele
R335Q	R335Q, R335W	9, 1 alleles
C361fs366stop	None	–
N372D	N372D, N372H	1, 1 alleles
R385stop	R385G, R385Q, R385stop	1, 2, 1 alleles

Potential alterations in *RGMC/HFE2/HJV* also are present in different cancers, but as with *RGMA* and *RGMB*, the possible functional consequences have not been determined. Predicted mutations (116, Table 5) have been identified at 102 different codons in 38 different neoplastic diseases, with the prevalence of these alterations ranging from 25% in prostate cancer, 10% in ovarian cancer, and 8.4% in melanoma, to 0.6% or less in colorectal carcinoma, salivary gland and renal cancer, leukemia, lymphomas, and others (see http://www.cbioportal.org/index.do?session_id=5b60fc90498eb8b3d5672fba). Putative amino acid substitutions or deletions predominated (106 different modifications at 92 locations; Table 5). Only 11 of these alterations were present in ExAC, with 9 having allelic frequencies of <0.002% (Table 5), and the others, a deletion or a duplication of Gly^69^, at 0.06 or 0.13%, respectively (Table 5). The other 10 changes consisted of premature stop codons and frame‐shifts, and except for Arg^385^ to stop codon were not found in ExAC (Table 5).

**Table 5:** Cancer‐associated mutations in RGMC/HJV

Mutation	Population variant	ExAC prevalence
G2V	None	–
P8L, P8S, P8T	None	–
G15D	None	–
L20I	None	–
T22N	None	–
L25I	None	–
L27M	None	–
L28I	None	–
L29I	None	–
S35F	None	–
I39T	None	–
R41C	R41C, R41L	1, 1 allele
V47L	None	–
A61E	None	–
G67E	None	–
G69del	G69del, G69dup	76, 154 alleles
Y86S	None	–
A94T	None	–
R95H	R95G	1 allele
D100E, D100N	D100H	1 allele
F103L	None	–
S105Ffs45stop	None	–
I110V	I110M	1 allele
D112Y	None	–
M114I	None	–
I115L, I115M	None	–
Q116K	None	–
N118Y	N118S	1 allele
Q122K	None	–
P129S	P129L	9 alleles
P133L	None	–
P136S	None	–
G141D	None	–
A144V	A144S, A144T	1, 1 allele
E151K	None	–
G159D	G159S	2 alleles
R160C, R160H	None	–
F164del	None	–
R176C, R176H	None	–
N196H, N196S	None	–
S206F	None	–
M208Wfs38stop	M208V, M208T, M208W	1, 1, 1 allele
A209V	None	–
L210S	None	–
T215I	T215A	1 allele
R218Q, R218W	None	–
T221S	None	–
K225N	None	–
M227T	None	–
I231V	I231T	2 alleles
E239Q	E239G	4 alleles
L243F	None	–
D249G	None	–
S251Y	None	–
G260E, G260R	None	–
S261Ifs9stop	None	–
S262G	None	–
L263F	None	–
S264L	S264L	2 alleles
Q266stop	None	–
N269K	N269S	1 allele
Y280N, Y280Hfs25stop, Y280Hfs31stop	None	–
R288Q	R288Q, R288W	1, 2 alleles
A305V	None	–
A307S	None	–
D313N	D313N	2 alleles
C317W	None	–
C321Vfs21stop	C321Y, C321W, C321stop	1, 2, 1 alleles
P323L	None	–
R329Q	R329L, R329P, R329Q, R329stop	2, 2, 2, 1 alleles
S330L	None	–
E331D	E331Q	1 allele
R332H	R332C, R332H	1, 5 alleles
N333K	N333S	1 allele
R334H	R334H	7 alleles
T339S	T339N	1 allele
I340T	None	–
R345W	R345Q, R345W	3, 1 alleles
K348N, K348R	None	–
E349K	None	–
S360F	None	–
S368Y	None	–
P371S	P371L	1 allele
F373C	None	–
A376E	None	–
A379T	A379E	1 allele
R385stop	R385G, R385Q, R385stop	1, 2, 1 alleles
L396F	None	–
P398L, P398S	None	–
D400V	None	–
A401V	None	–
G402E	None	–
V403A	V403I	1 allele
S406F	None	–
L415F, L415H	None	–
S416F, S416Y	S416P	2 alleles
L421M	None	–
W422stop	W422C	1 allele
L423I	None	–
I425T	None	–
Q426stop	None	–

## Discussion

Information extracted from publically available databases has been collected and then analyzed here to gain insights into the genomics and population genetics of the RGM family in humans. Results identify extensive variation in gene expression patterns, substantial alternative RNA splicing, and a range of possible missense alterations and other modifications in the coding regions of each of the three genes studied, which were not apparent previously, and in many cases are detected in individuals with different types of cancers (Tables 2, 3, 5). In addition, the data show that selected amino acid substitutions are highly prevalent in the world's population, with minor allele frequencies of up to 37% for RGMA and up to 8% for RGMB (Fig. 5). Collectively, these results indicate that protein sequence variation is common in the human RGM family, as has been observed for some other human proteins (Rotwein 2017a,b), and it thus appears likely that these variants could have a significant population impact on human physiology and/or disease predisposition.

### RGMA and RGMB: genes, mRNAs, and proteins

By combining information from the Ensembl and UCSC Genome Browsers with data extracted from GTEx, complex patterns of expression have been elucidated here for each human *RGM* gene, particularly in the distribution of different mRNA isoforms (Figs. 2, 3, 4). For example, these results now demonstrate that both *RGMA* and *RGMB* genes are widely expressed in many different adult human organs and tissues, with most of the transcripts encoding one of the several “full‐length” proteins, as differences among these isoforms are found primarily at the NH_2_‐terminus in the presumptive signal peptides (Figs. 2, 3). Although a few studies have examined possible effects of RGMA or RGMB in humans (Demicheva et al. 2015; Shi et al. 2015; Li et al. 2016; Muller et al. 2016), most publications to date have focused on experimental model systems (Matsunaga et al. 2004, 2006; Niederkofler et al. 2004; Rajagopalan et al. 2004; Samad et al. 2004; Hata et al. 2006; Tanabe and Yamashita 2014). Thus, these new observations will provide opportunities to develop new insights into *RGMA* and *RGMB* gene regulation and their protein functions in a variety of human physiological and pathological processes. Of particular note here is the fact that according to GTEx both *RGMA* and *RGMB* are expressed at similarly high transcript levels in the muscularis region of the esophagus, and within the gastro‐esophageal junction (Figs. 2C, 3C, and not shown), raising the question of whether either or both proteins might be involved in aspects of smooth muscle function, such as its coordination by the sympathetic and parasympathetic nervous systems or other signals during swallowing or digestion of food (Woodland et al. 2013). As mRNAs encoding neogenin (*NEO1*) and BMP receptors (*BMPR1A*,* BMPR1B*, and *BMPR2*) also are expressed in these parts of the esophagus, it is conceivable that different RGM‐mediated signaling pathways could be active in different parts of this organ.

Another surprising observation with regard to *RGMA* and *RGMB* is their expression in a range of different cancers, with transcripts encoding mutant proteins being detected in up to 10% of cases of prostate cancer (*RGMB*) and in 3.5% of ovarian carcinomas (*RGMA*, see Results), again providing evidence for their unexplored roles in human disease. As the majority of these predicted mutations were found to be rare in the general population used in ExAC (although nearly all of the most highly prevalent amino acid substitution alleles were present; see Tables 2 and 3), these data argue for possible pathophysiological actions for RGMA and RGMB in human neoplasms, and represent another illustration in which focused analysis of information extracted from large‐scale databases can help identify new areas of investigation with possible biomedical consequences.

### The special case of RGMC/HFE2/HJV

Data collected and assessed from Ensembl, the UCSC Genome Browser, and GTEx also have revealed some unexpected aspects of human *RGMC/HFE2/HJV* gene expression (Fig. 4). Even though restriction of transcripts to skeletal muscle, liver, and heart had been recognized previously (Kuninger et al. 2004; Papanikolaou et al. 2004; Schmidtmer and Engelkamp 2004), remarkably it now appears that only ~20% of *RGMC/HFE2/HJV* mRNAs found in human tissues encode the 426‐amino acid full‐length protein (Fig. 4D). The other mRNAs, which comprise the vast majority of transcripts in each tissue type (80 to 90%, Fig. 4D), encode proteins that are truncated at the NH_2_‐terminus. These latter species lack most of the N‐RGM domain (313‐residue isoform), all of the N‐RGM segment and the entire von Willebrand factor type D domain (200‐amino acid protein), or all but 93‐amino acids in the center of the molecule (Figs. 4B, 5C). The observations also raise questions regarding which of these variant RGMC/HJV proteins are biologically active molecules, and what are their presumptive activities. In animal and cell‐based studies, several different‐length versions of RGMC/HJV have been noted, but these have been characterized as being derived from differential protein processing during biosynthesis, and from proteolytic cleavage of the mature GPI‐linked cell surface molecule either by pro‐protein convertases such as furin (Kuninger et al. 2006, 2008; Silvestri et al. 2008a), or by the serine protease, matriptase‐2 (Silvestri et al. 2008b). Thus, these new observations, which have resulted from analyses of information in databases, define a potentially novel and alternative way that different RGMC/HJV protein isoforms are produced in humans.

Unlike what is observed for RGMA and RGMB, presumptive RGMC/HJV protein variants within the ExAC population are very uncommon, collectively occurring in <1.5% of 60,706 genomes versus 86% for RGMA and 9% for RGMB (Fig. 5). Moreover, even though 17 of 43 amino acid substitution, frame‐shift, and stop codon mutations associated with juvenile hemochromatosis have been found in the ExAC study cohort, only a single disease‐associated allele is present in more than 0.025% of the population (Ala^310^ to Gly, at ~0.7%), and 13 are represented just 1–3 times in the 121,412 ExAC alleles (Table 4). This result suggests that any possible contribution of *RGMC*/*HFE2/HJV* heterozygosity toward iron overload in the general population is minimal, in marked contrast to the high prevalence of HFE protein variants, at least in European‐derived groups (Barton et al. 2015; Wallace and Subramaniam 2016).

As seen for RGMA and RGMB, predicted mutations of RGMC/HJV are found in many different cancers, with transcripts encoding mutant proteins being detected in 25% of prostate cancers, 10% of ovarian carcinomas, and 8.4% of melanomas (see Results). Remarkably, both prostate and ovarian cancers are the diseases in which mutant RGMB and RGMA molecules also have been found at highest prevalence, respectively (see Results and above). Moreover, only ~10% of the 106 different mutations in RGMC/HJV detected in cancers are present in ExAC, with all but one of them being rare (found fewer than 5 times) in the 121,412 alleles studied (Table 5).

### Limitations and strengths of population‐based sequence data for understanding RGM actions

As with any large‐scale DNA or RNA‐based sequencing project, ExAC and GTEx respectively contain the potential materials for new biological and biomedical applications, as well as errors and ambiguities. From the perspective of the three *RGM* family genes, potential problems include the choice of minor transcripts as the reference sequences for proteins. This is especially true for RGMA, in which the mRNA species encoding the 458‐amino acid protein isoform selected by ExAC (see Table 1) appears to comprise ≤2% of transcripts in human organs and tissues in GTEx (isoform 5, Fig. 2D). In contrast, for RGMB, the predominant transcript in 9 of the 10 tissues surveyed in GTEx encodes the major 478‐residue protein species (all but testes, Fig. 3D). Another complication here is the potential variation in RNA quality in GTEx samples, especially since both the time from tissue harvesting to RNA extraction and the methods employed to isolate RNA are unknown. It thus seems possible that transcript degradation may skew the results seen in GTEx RNA‐sequencing libraries derived from at least some of the different organs and tissues. Furthermore, as the population distribution of the GTEx dataset is unknown, there are no data to determine whether or not expression of different mRNA isoforms varies among different groups, perhaps in conjunction with population‐specific DNA polymorphisms (Khera et al. 2018; Yengo et al. 2018). Other limitations that could contribute to problems in data interpretation include the potential non‐representative nature of the ExAC study population, as over 60% of samples are derived from European individuals, with ~20% from South or East Asians, and only ~8% each from Hispanic or African groups (Lek et al. 2016). Thus, the actual rate and potential extent of variation among RGM proteins has not been established fully yet, and could change once exome sequencing data are obtained from more individuals and are expanded to include larger numbers of people from different human population groups. Moreover, there is an undefined but probable error rate associated with nucleotide changes that appear only once or just a few times in the 121,412 ExAC chromosomes studied.

Despite these challenges and difficulties, the data in ExAC, GTEx, and in the various cancer medicine portals examined here, provide potentially exciting new opportunities to evaluate contributes of the RGM family, and RGMA and RGMB in particular, to human physiology and disease. Since *RGMA* and *RGMB* are expressed in the vast majority of adult human organs and tissues (48 of 51 for *RGMA* and 49 of 51 for *RGMB*), the encoded proteins are likely to be involved in some regulatory processes. Perhaps immune cell function is in one of these areas, since RGMA is expressed in dendritic cells and neogenin is found in CD4 +  T lymphocytes (Muramatsu et al. 2011).

Modern human populations represent the outcomes of many interactions over long time frames with different ancestral groups. Not only do the DNA marks in our genomes derived from extinct populations such as Neanderthals, Denisovans, and others document these past relationships (Jones et al. 2015; Vattathil and Akey 2015; Clarkson et al. 2017; Hublin et al. 2017), but some of the introgressed DNA continues to influence human physiology or disease susceptibility to the present day (Dannemann and Kelso 2017; Prufer et al. 2017). Opportunities abound to use the data in ExAC, GTEx, and other large‐scale population‐based repositories such as the British Biobank (Khera et al. 2018; Yengo et al. 2018) as the springboard toward developing novel and medically important research questions with high biological and biomedical significance.

## Conflict of Interest

The author has no perceived or potential conflict of interest, financial or otherwise.

## References

[phy213959-bib-0001] Acuna‐Hidalgo, R. , J. A. Veltman , and A. Hoischen . 2016 New insights into the generation and role of de novo mutations in health and disease. Genome Biol. 17:241–260. 27894357 10.1186/s13059-016-1110-1 PMC5125044

[phy213959-bib-0002] Babitt, J. L. , Y. Zhang , T. A. Samad , Y. Xia , J. Tang , J. A. Campagna , et al. 2005 Repulsive guidance molecule (RGMa), a DRAGON homologue, is a bone morphogenetic protein co‐receptor. J. Biol. Chem. 280:29820–29827. 15975920 10.1074/jbc.M503511200

[phy213959-bib-0003] Bahcall, O. G. 2016 Genetic variation: ExAC boosts clinical variant interpretation in rare diseases. Nat. Rev. Genet. 17:584. 27629930 10.1038/nrg.2016.121

[phy213959-bib-0004] Barton, J. C. , C. Q. Edwards , and R. T. Acton . 2015 HFE gene: structure, function, mutations, and associated iron abnormalities. Gene 574:179–192. 26456104 10.1016/j.gene.2015.10.009 PMC6660136

[phy213959-bib-0005] Battle, A. , C. D. Brown , B. E. Engelhardt , and S. B. Montgomery . 2017 Genetic effects on gene expression across human tissues. Nature 550:204–213. 29022597 10.1038/nature24277 PMC5776756

[phy213959-bib-0006] Bernet, A. , and P. Mehlen . 2007 Dependence receptors: when apoptosis controls tumor progression. Bull. Cancer 94:E12–E17. 17449433

[phy213959-bib-0007] Cerami, E. , J. Gao , U. Dogrusoz , B. E. Gross , S. O. Sumer , B. A. Aksoy , et al. 2012 The cBio cancer genomics portal: an open platform for exploring multidimensional cancer genomics data. Cancer Discov. 2:401–404. 22588877 10.1158/2159-8290.CD-12-0095 PMC3956037

[phy213959-bib-0008] Clarkson, C. , Z. Jacobs , B. Marwick , R. Fullagar , L. Wallis , M. Smith , et al. 2017 Human occupation of northern Australia by 65,000 years ago. Nature 547:306–310. 28726833 10.1038/nature22968

[phy213959-bib-0009] Conrad, S. , H. Genth , F. Hofmann , I. Just , and T. Skutella . 2007 Neogenin‐RGMa signaling at the growth cone Is BMP‐independent and involves RhoA, rock and PKC. J. Biol. Chem. 282:16423–16433. 17389603 10.1074/jbc.M610901200

[phy213959-bib-0010] Core, A. B. , S. Canali , and J. L. Babitt . 2014 Hemojuvelin and bone morphogenetic protein (BMP) signaling in iron homeostasis. Front. Pharmacol. 5:104 (e collection). 24860505 10.3389/fphar.2014.00104 PMC4026703

[phy213959-bib-0011] Corradini, E. , J. L. Babitt , and H. Y. Lin . 2009 The RGM/DRAGON family of BMP co‐receptors. Cytokine Growth Factor Rev. 20:389–398. 19897400 10.1016/j.cytogfr.2009.10.008 PMC3715994

[phy213959-bib-0012] Dannemann, M. , and J. Kelso . 2017 The contribution of Neanderthals to phenotypic variation in modern humans. Am. J. Hum. Genet. 101:578–589. 28985494 10.1016/j.ajhg.2017.09.010 PMC5630192

[phy213959-bib-0013] Demicheva, E. , Y. F. Cui , P. Bardwell , S. Barghorn , M. Kron , A. H. Meyer , et al. 2015 Targeting repulsive guidance molecule A to promote regeneration and neuroprotection in multiple sclerosis. Cell Rep. 10:1887–1898. 25801027 10.1016/j.celrep.2015.02.048

[phy213959-bib-0014] Gao, J. , B. A. Aksoy , U. Dogrusoz , G. Dresdner , B. Gross , S. O. Sumer , et al. 2013 Integrative analysis of complex cancer genomics and clinical profiles using the cBioPortal. Sci. Signal. 6:l1. 10.1126/scisignal.2004088 PMC4160307 23550210

[phy213959-bib-0015] Gehrke, S. G. , A. Pietrangelo , M. Kascak , A. Braner , M. Eisold , H. Kulaksiz , et al. 2005 HJV gene mutations in European patients with juvenile hemochromatosis. Clin. Genet. 67:425–428. 15811010 10.1111/j.1399-0004.2005.00413.x

[phy213959-bib-0016] eGTEx Project . 2017 Enhancing GTEx by bridging the gaps between genotype, gene expression, and disease. Nat. Genet. 49:1664–1670. 29019975 10.1038/ng.3969 PMC6636856

[phy213959-bib-0017] Hata, A. , and Y. G. Chen . 2016 TGF‐beta signaling from receptors to Smads. Cold Spring Harb. Perspect. Biol. 8:1–31 (e collection). 10.1101/cshperspect.a022061 PMC5008074 27449815

[phy213959-bib-0018] Hata, K. , M. Fujitani , Y. Yasuda , H. Doya , T. Saito , S. Yamagishi , et al. 2006 RGMa inhibition promotes axonal growth and recovery after spinal cord injury. J. Cell Biol. 173:47–58. 16585268 10.1083/jcb.200508143 PMC2063787

[phy213959-bib-0019] Huang, F. W. , J. L. Pinkus , G. S. Pinkus , M. D. Fleming , and N. C. Andrews . 2005 A mouse model of juvenile hemochromatosis. J. Clin. Invest. 115:2187–2191. 16075059 10.1172/JCI25049 PMC1180543

[phy213959-bib-0020] Hublin, J. J. , A. Ben‐Ncer , S. E. Bailey , S. E. Freidline , S. Neubauer , M. M. Skinner , et al. 2017 New fossils from Jebel Irhoud, Morocco and the pan‐African origin of Homo sapiens. Nature 546:289–292. 28593953 10.1038/nature22336

[phy213959-bib-0021] Jones, E. R. , G. Gonzalez‐Fortes , S. Connell , V. Siska , A. Eriksson , R. Martiniano , et al. 2015 Upper Palaeolithic genomes reveal deep roots of modern Eurasians. Nat. Commun. 6:8912–8919. 26567969 10.1038/ncomms9912 PMC4660371

[phy213959-bib-0022] Karczewski, K. J. , B. Weisburd , B. Thomas , M. Solomonson , D. M. Ruderfer , D. Kavanagh , et al. 2017 The ExAC browser: displaying reference data information from over 60 000 exomes. Nucleic Acids Res. 45:D840–D845. 27899611 10.1093/nar/gkw971 PMC5210650

[phy213959-bib-0023] Katsanis, N. 2016 The continuum of causality in human genetic disorders. Genome Biol. 17:233–237. 27855690 10.1186/s13059-016-1107-9 PMC5114767

[phy213959-bib-0024] Keino‐Masu, K. , M. Masu , L. Hinck , E. D. Leonardo , S. S. Chan , J. G. Culotti , et al. 1996 Deleted in Colorectal Cancer (DCC) encodes a netrin receptor. Cell 87:175– 185. 8861902 10.1016/s0092-8674(00)81336-7

[phy213959-bib-0025] Khera, A. V. , M. Chaffin , K. G. Aragam , M. E. Haas , C. Roselli , S. H. Choi , et al. 2018 Genome‐wide polygenic scores for common diseases identify individuals with risk equivalent to monogenic mutations. Nat. Genet. 50:1219–1224. 30104762 10.1038/s41588-018-0183-z PMC6128408

[phy213959-bib-0026] Kuninger, D. , R. Kuzmickas , B. Peng , J. E. Pintar , and P. Rotwein . 2004 Gene discovery by microarray: identification of novel genes induced during growth factor‐mediated muscle cell survival and differentiation. Genomics 84:876–889. 15475267 10.1016/j.ygeno.2004.07.013

[phy213959-bib-0027] Kuninger, D. , R. Kuns‐Hashimoto , R. Kuzmickas , and P. Rotwein . 2006 Complex biosynthesis of the muscle‐enriched iron regulator RGMc. J. Cell Sci. 119:3273–3283. 16868025 10.1242/jcs.03074

[phy213959-bib-0028] Kuninger, D. , R. Kuns‐Hashimoto , M. Nili , and P. Rotwein . 2008 Pro‐protein convertases control the maturation and processing of the iron‐regulatory protein, RGMc/hemojuvelin. BMC Biochem. 9:1–9. 18384687 10.1186/1471-2091-9-9 PMC2323002

[phy213959-bib-0029] Kuns‐Hashimoto, R. , D. Kuninger , M. Nili , and P. Rotwein . 2008 Selective binding of RGMc/hemojuvelin, a key protein in systemic iron metabolism, to BMP‐2 and neogenin. Am. J. Physiol. Cell Physiol. 294:C994–C1003. 18287331 10.1152/ajpcell.00563.2007

[phy213959-bib-0030] Lanzara, C. , A. Roetto , F. Daraio , S. Rivard , R. Ficarella , H. Simard , et al. 2004 Spectrum of hemojuvelin gene mutations in 1q‐linked juvenile hemochromatosis. Blood 103:4317–4321. 14982873 10.1182/blood-2004-01-0192

[phy213959-bib-0031] Lebreton, S. , C. Zurzolo , and S. Paladino . 2018 Organization of GPI‐anchored proteins at the cell surface and its physiopathological relevance. Crit. Rev. Biochem. Mol. Biol. 53:403–419. 30040489 10.1080/10409238.2018.1485627

[phy213959-bib-0032] Lek, M. , K. J. Karczewski , E. V. Minikel , K. E. Samocha , E. Banks , T. Fennell , et al. 2016 Analysis of protein‐coding genetic variation in 60,706 humans. Nature 536:285–291. 27535533 10.1038/nature19057 PMC5018207

[phy213959-bib-0033] Leonardo, E. D. , L. Hinck , M. Masu , K. Keino‐Masu , S. L. Ackerman , and M. Tessier‐Lavigne . 1997 Vertebrate homologues of *C. elegans* UNC‐5 are candidate netrin receptors. Nature 386:833–838. 9126742 10.1038/386833a0

[phy213959-bib-0034] Li, J. , L. Ye , X. Shi , J. Chen , F. Feng , Y. Chen , et al. 2016 Repulsive guidance molecule B inhibits metastasis and is associated with decreased mortality in non‐small cell lung cancer. Oncotarget 7:15678–15689. 26910889 10.18632/oncotarget.7463 PMC4941269

[phy213959-bib-0035] Matsunaga, E. , S. Tauszig‐Delamasure , P. P. Monnier , B. K. Mueller , S. M. Strittmatter , P. Mehlen , et al. 2004 RGM and its receptor neogenin regulate neuronal survival. Nat. Cell Biol. 6:749–755. 15258591 10.1038/ncb1157

[phy213959-bib-0036] Matsunaga, E. , H. Nakamura , and A. Chedotal . 2006 Repulsive guidance molecule plays multiple roles in neuronal differentiation and axon guidance. J. Neurosci. 26:6082–6088. 16738252 10.1523/JNEUROSCI.4556-05.2006 PMC6675224

[phy213959-bib-0037] Mehlen, P. , and L. Mazelin . 2003 The dependence receptors DCC and UNC5H as a link between neuronal guidance and survival. Biol. Cell 95:425–436. 14597260 10.1016/s0248-4900(03)00072-8

[phy213959-bib-0038] Monnier, P. P. , A. Sierra , P. Macchi , L. Deitinghoff , J. S. Andersen , M. Mann , et al. 2002 RGM is a repulsive guidance molecule for retinal axons. Nature 419:392–395. 12353034 10.1038/nature01041

[phy213959-bib-0039] Morikawa, M. , R. Derynck , and K. Miyazono . 2016 TGF‐beta and the TGF‐beta family: context‐dependent roles in cell and tissue physiology. Cold Spring Harb. Perspect. Biol. 8:1–24 (e collection). 10.1101/cshperspect.a021873 PMC4852809 27141051

[phy213959-bib-0040] Muller, T. , I. Trommer , S. Muhlack , and B. K. Mueller . 2016 Levodopa increases oxidative stress and repulsive guidance molecule A levels: a pilot study in patients with Parkinson's disease. J. Neural Transm. (Vienna) 123:401–406. 26880022 10.1007/s00702-016-1519-4

[phy213959-bib-0041] Muramatsu, R. , T. Kubo , M. Mori , Y. Nakamura , Y. Fujita , T. Akutsu , et al. 2011 RGMa modulates T cell responses and is involved in autoimmune encephalomyelitis. Nat. Med. 17:488–494. 21423182 10.1038/nm.2321

[phy213959-bib-0042] Niederkofler, V. , R. Salie , M. Sigrist , and S. Arber . 2004 Repulsive guidance molecule (RGM) gene function is required for neural tube closure but not retinal topography in the mouse visual system. J. Neurosci. 24:808–818. 14749425 10.1523/JNEUROSCI.4610-03.2004 PMC6729817

[phy213959-bib-0043] Niederkofler, V. , R. Salie , and S. Arber . 2005 Hemojuvelin is essential for dietary iron sensing, and its mutation leads to severe iron overload. J. Clin. Invest. 115:2180–2186. 16075058 10.1172/JCI25683 PMC1180556

[phy213959-bib-0044] Papanikolaou, G. , M. E. Samuels , E. H. Ludwig , M. L. MacDonald , P. L. Franchini , M. P. Dube , et al. 2004 Mutations in HFE2 cause iron overload in chromosome 1q‐linked juvenile hemochromatosis. Nat. Genet. 36:77– 82. 14647275 10.1038/ng1274

[phy213959-bib-0045] Prufer, K. , C. de Filippo , S. Grote , F. Mafessoni , P. Korlevic , M. Hajdinjak , et al. 2017 A high‐coverage Neandertal genome from Vindija Cave in Croatia. Science 358:655–658. 28982794 10.1126/science.aao1887 PMC6185897

[phy213959-bib-0046] Quintana‐Murci, L. 2016 Understanding rare and common diseases in the context of human evolution. Genome Biol. 17:225–239. 27821149 10.1186/s13059-016-1093-y PMC5098287

[phy213959-bib-0047] Rajagopalan, S. , L. Deitinghoff , D. Davis , S. Conrad , T. Skutella , A. Chedotal , et al. 2004 Neogenin mediates the action of repulsive guidance molecule. Nat. Cell Biol. 6:756–762. 15258590 10.1038/ncb1156

[phy213959-bib-0048] Rotwein, P. 2017a The new genomics: what molecular databases can tell us about human population variation and endocrine disease. Endocrinology 158:2035–2042. 28498917 10.1210/en.2017-00338 PMC7282473

[phy213959-bib-0049] Rotwein, P. 2017b Large‐scale analysis of variation in the insulin‐like growth factor family in humans reveals rare disease links and common polymorphisms. J. Biol. Chem. 292:9252–9261. 28389567 10.1074/jbc.M117.783639 PMC5454106

[phy213959-bib-0050] Ruderfer, D. M. , T. Hamamsy , M. Lek , K. J. Karczewski , D. Kavanagh , K. E. Samocha , et al. 2016 Patterns of genic intolerance of rare copy number variation in 59,898 human exomes. Nat. Genet. 48:1107–1111. 27533299 10.1038/ng.3638 PMC5042837

[phy213959-bib-0051] Samad, T. A. , A. Srinivasan , L. A. Karchewski , S. J. Jeong , J. A. Campagna , R. R. Ji , et al. 2004 DRAGON: a member of the repulsive guidance molecule‐related family of neuronal‐ and muscle‐expressed membrane proteins is regulated by DRG11 and has neuronal adhesive properties. J. Neurosci. 24:2027–2036. 14985445 10.1523/JNEUROSCI.4115-03.2004 PMC6730385

[phy213959-bib-0052] Samad, T. A. , A. Rebbapragada , E. Bell , Y. Zhang , Y. Sidis , S. J. Jeong , et al. 2005 DRAGON, a bone morphogenetic protein co‐receptor. J. Biol. Chem. 280:14122–14129. 15671031 10.1074/jbc.M410034200

[phy213959-bib-0053] Schmidtmer, J. , and D. Engelkamp . 2004 Isolation and expression pattern of three mouse homologues of chick Rgm. Gene Expr. Patterns 4:105–110. 14678836 10.1016/s1567-133x(03)00144-3

[phy213959-bib-0054] Severyn, C. J. , U. Shinde , and P. Rotwein . 2009 Molecular biology, genetics and biochemistry of the repulsive guidance molecule family. Biochem J. 422:393–403. 19698085 10.1042/BJ20090978 PMC4242795

[phy213959-bib-0055] Shi, Y. , G. B. Chen , X. X. Huang , C. X. Xiao , H. H. Wang , Y. S. Li , et al. 2015 Dragon (repulsive guidance molecule b, RGMb) is a novel gene that promotes colorectal cancer growth. Oncotarget 6:20540–20554. 26029998 10.18632/oncotarget.4110 PMC4653024

[phy213959-bib-0056] Siebold, C. , T. Yamashita , P. P. Monnier , B. K. Mueller , and R. J. Pasterkamp . 2017 RGMs: structural insights, molecular regulation, and downstream signaling. Trends Cell Biol. 27:365–378. 28007423 10.1016/j.tcb.2016.11.009 PMC5404723

[phy213959-bib-0057] Silvestri, L. , A. Pagani , and C. Camaschella . 2008a Furin‐mediated release of soluble hemojuvelin: a new link between hypoxia and iron homeostasis. Blood 111:924–931. 17938254 10.1182/blood-2007-07-100677

[phy213959-bib-0058] Silvestri, L. , A. Pagani , A. Nai , I. De Domenico , J. Kaplan , and C. Camaschella . 2008b The serine protease matriptase‐2 (TMPRSS6) inhibits hepcidin activation by cleaving membrane hemojuvelin. Cell Metab. 8:502–511. 18976966 10.1016/j.cmet.2008.09.012 PMC2648389

[phy213959-bib-0059] Tanabe, S. , and T. Yamashita . 2014 Repulsive guidance molecule‐a is involved in Th17‐cell‐induced neurodegeneration in autoimmune encephalomyelitis. Cell Rep. 9:1459–1470. 25456136 10.1016/j.celrep.2014.10.038

[phy213959-bib-0060] Vattathil, S. , and J. M. Akey . 2015 Small amounts of archaic admixture provide big insights into human history. Cell 163:281–284. 26451479 10.1016/j.cell.2015.09.042

[phy213959-bib-0061] Wallace, D. F. , and V. N. Subramaniam . 2016 The global prevalence of HFE and non‐HFE hemochromatosis estimated from analysis of next‐generation sequencing data. Genet. Med. 18:618–626. 26633544 10.1038/gim.2015.140

[phy213959-bib-0062] Woodland, P. , D. Sifrim , A. L. Krarup , C. Brock , J. B. Frokjaer , C. Lottrup , et al. 2013 The neurophysiology of the esophagus. Ann. N. Y. Acad. Sci. 1300:53–70. 24117634 10.1111/nyas.12238

[phy213959-bib-0063] Xia, Y. , J. L. Babitt , Y. Sidis , R. T. Chung , and H. Y. Lin . 2008 Hemojuvelin regulates hepcidin expression via a selective subset of BMP ligands and receptors independently of neogenin. Blood 111:5195–5204. 18326817 10.1182/blood-2007-09-111567 PMC2384142

[phy213959-bib-0064] Yang, F. , A. P. J. West , G. P. Allendorph , S. Choe , and P. J. Bjorkman . 2008 Neogenin interacts with hemojuvelin through its two membrane‐proximal fibronectin type III domains. Biochemistry 47:4237–4245. 18335997 10.1021/bi800036h PMC2819367

[phy213959-bib-0065] Yengo, L. , J. Sidorenko , K. E. Kemper , Z. Zheng , A. R. Wood , M. N. Weedon , et al. 2018 Meta‐analysis of genome‐wide association studies for height and body mass index in approximately 700000 individuals of European ancestry. Hum. Mol. Genet. 27:3641–3649. 30124842 10.1093/hmg/ddy271 PMC6488973

